# Sub-axial cervical spine injuries: Modified Stellerman’s algorithm

**DOI:** 10.4103/0019-5413.80046

**Published:** 2011

**Authors:** Arjun Shetty, Abhishek R Kini, Deepak Muthappa

**Affiliations:** Department of Neurosurgery, Kasturba Medical College, Manipal, and Consultant Neurosurgeon, Tejasvini Hospital and SSIOT, Kadri, India; 1 Department of Orthopaedics and Traumatology, Tejasvini Hospital and SSIOT, Kadri, India; 2 K.S.Hegde Medical Academy, Mangalore, India

**Keywords:** Anterior decompression and fusion, sub axial spine injuries, Stellerman’s algorithm

## Abstract

**Background:**

Global fusion is recommended in sub-axial cervical spine injuries with retrolisthesis, translation rotation injuries associated with end plate or tear drop fractures. We propose a modification of Stellerman’s algorithm which we have used where in patients are primarily treated via anterior decompression and fixation. Global fusion was done only in cases where post-decompression traction does not achieve reduction in cases with locked facets.

**Materials and Methods:**

Two hundred and thirty consecutive patients with sub-axial cervical spine injuries were studied in a prospective trial over a 7 year period. Seven cases with posterior compression alone were not subjected to our protocol. Of the other 223 cases, 191 cases who on radiological evaluation needed surgery were initially approached anteriorly. Decompression was effected through a corpectomy in 14 cases and a single or multiple level disc excisions were performed in the others. Cases with cervical listhesis (n=36) where on table reduction could not be achieved following decompression were subjected to progressive skeletal traction for 48 h. Posterior facetectomy and global fixation was done for patients in whom reduction could not be achieved despite post-decompression traction (n=11).

**Results:**

Of the 223 cases, 20 cases were managed conservatively, 12 cases expired pre-operatively, and the remaining 191 cases needed surgical intervention. Out of the 154 cases of distraction/rotation/translation injuries on table reduction could be achieved in 118 cases (76.6%). Thirty-six patients had locked facets (23 cases were bifacetal, 13 cases unifacetal) and of these 36 cases reduction could be achieved with post-anterior decompression traction in 25 patients (16.2%); however, only 11 cases (7.1%)–8 bifacetal and 3 unifacetal dislocations–needed posterior facetectomy and global fusion. One hundred and forty-three patients were followed up for a minimum period of 6 months. One hundred and twenty-six patients showed evidence of complete fusion (88.1%) while the remaining 17 (11.8) showed evidence of partial fusion. There were no signs of instability on clinical and radiological evaluation in any of the cases. Reduction of graft height was noted in 18 patients (12.5%). There were eight cases of immediate postoperative mortality and two cases of delayed mortality in our series of cases.

**Conclusion:**

We feel that on table decompression and reduction followed by anterior stabilization can be used as the initial surgical approach to manage most types of cervical injuries. In rotation/translational cases where reduction cannot be achieved, monitored cervical traction on the decompressed spine can safely achieve reduction and hence avoid the need for a posterior facetectomy in a large percentage of cases.

## INTRODUCTION

**Figure 1:**
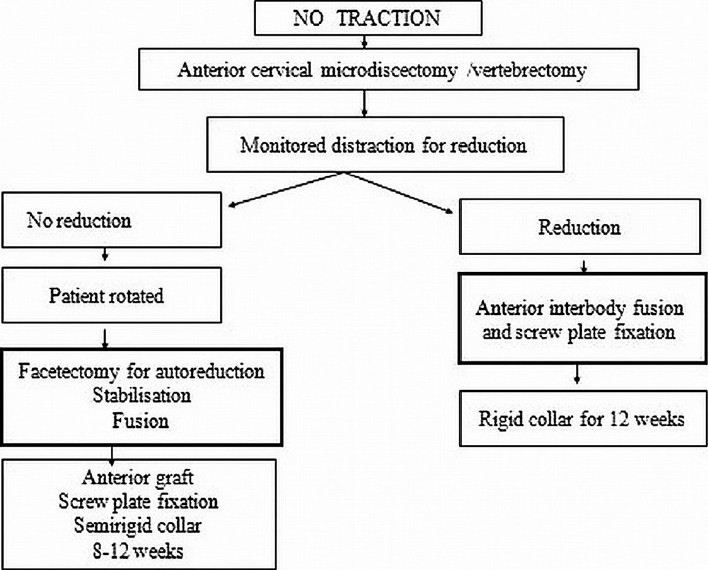
Stellerman’s algorithm for bifacetal subluxations

The Stellerman’s algorithm^1^ uses the anterior approach for decompression, reduction, and fixation of cervical spine injuries with facetal dislocation. In cases where on table reduction was not achieved (locked facets), posterior facetectomy with global fixation is proposed [Figure 1]. In cases with retrolisthesis and translation rotational injuries associated with end plate or tear drop fractures, various authors have recommended global fixation.

In our protocol all cases of sub-axial cervical spine injuries, including those mentioned above were initially managed with an anterior decompression, fusion, and fixation. In cases with locked facets, instead of immediate posterior facetectomy and global fusion as advocated by Stellerman’s algorithm,^1^ we have used post-decompression traction to achieve reduction in a significant number of patients and restricted the need for a posterior facetectomy and global fusion to those patients in whom post-decompression traction did not achieve reduction.

## MATERIALS AND METHODS

Two hundred and thirty consecutive patients were studied. Seven patients, three with depressed laminar fractures and four with posteriorly situated epidural hematomas were subjected to laminoplasty/laminectomy and posterior stabilization primarily and these patients were not subjected to our protocol. The remaining 223 patients were managed as per our protocol. All patients were subjected to a detailed neurological evaluation and graded on the ASIA (American spinal injury association) scale.^2^ Magnetic resonance imaging (MRI) scans were routinely asked for as part of our protocol. CT (computerized tomography) scans done were patients who had vertebral body fracture and/or facetal fractures. This study was approved by the institutional Ethics Committee.

**Figure 2:**
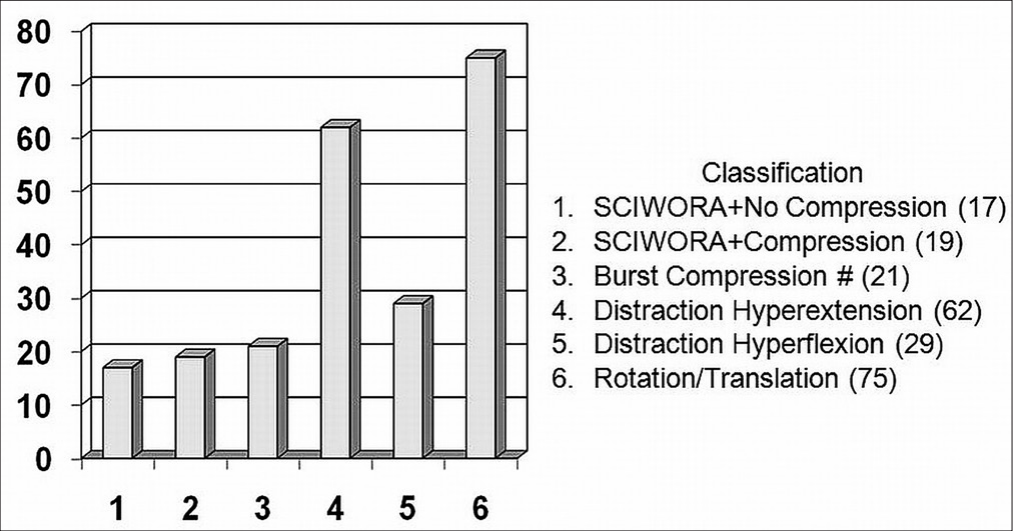
Bar chart showing distribution of cases

**Figure 3:**
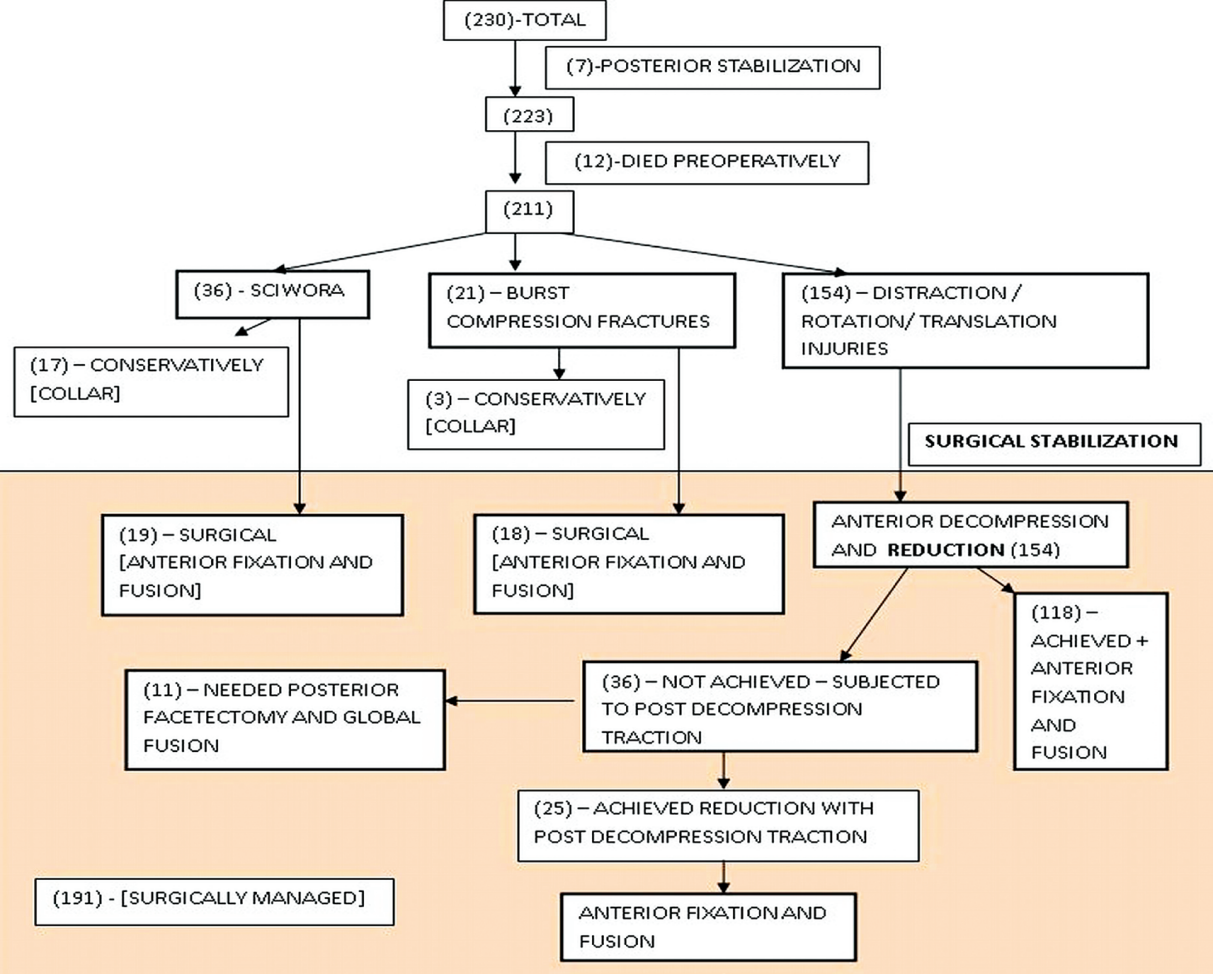
Flowchart of our cases with their numbers and treatment protocol

Patients were classified into five groups: [Figures 2 and 3] (a) Sciwora (36 cases), (b) compression or burst fractures (21 cases), (c) distraction hyperextension (62 cases), (d) distraction hyperflexion (29 cases), (e) rotation translation (75 cases).

Twelve patients presented with severe pain respiratory difficulty and or associated polytrauma and expired shortly after admission (5–distraction hyperextension, 2–distraction hyperflexion, and 5–rotation translation).

**Figure 4:**
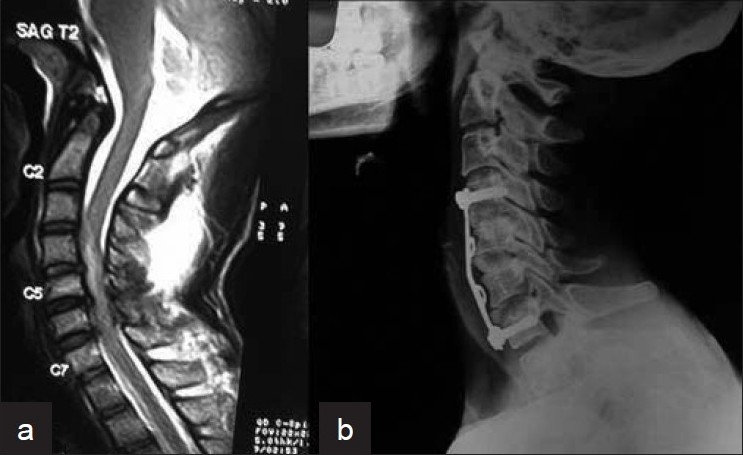
(a) Sagittal MRI T2WI of cervical spine in a posttraumatic cervical spine injury showing three level disc herniation. (b) 16 months post-operative X-ray cervical spine (lateral view) after excision, fusion and fixation showing good bony fusion

Of the 36 patients with the normal x-rays and neurological deficits (SCIWORA), 17 were found to have no evidence of instability or neurological compression on MRI. These patients were treated conservatively with a Philadelphia collar for 6 weeks. The remaining 19 cases showed evidence of cord or root compression due to a prolapsed disc fragment. Compression was at one level in 16 cases, two level in 2 cases and three level in 1 case [Figure 4]. In all cases, disc excision and fusion with iliac graft with fixation using screws and plate were done.

Eighteen patients with burst compression fractures were subjected to corpectomy and fusion followed by anterior fixation using plate and screws. Of these, one patient needed a three-level corpectomy, two patients needed a two-level corpectomy, and the remaining (15 cases) needed single-level corpectomy. In all these patients, fusion was achieved using autogenous iliac crest graft except in one child who needed a single level corpectomy, where the fibular graft was used. Three cases where the compression was minimal and there was no neural canal compression were managed conservatively using a Halo brace for 6 weeks.

All other patients of distraction or rotation translation injury (154) initially underwent micro discectomy to ensure the decompression of the neural canal. In eighteen cases drilling of the small area of the upper or lower vertebral body was required to extract the prolapsed disc fragment. Following decompression on table reduction under anesthesia with skeletal traction was attempted applying linear traction. In 118 cases, reduction was achieved on table and these patients were then subjected to iliac graft fusion and fixation with cervical plate and screws.

**Figure 5:**
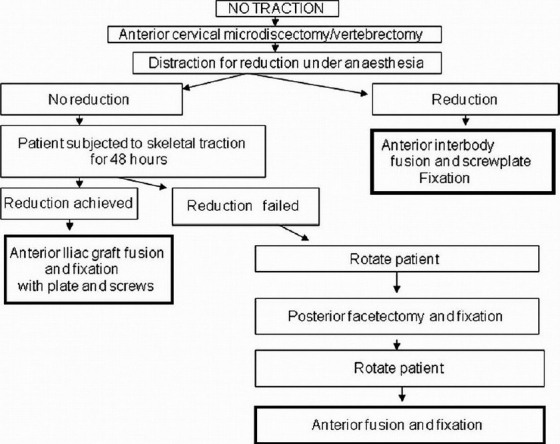
Proposed algorithm of management of sub-axial cervical spine injuries

In 36 patients on table reduction could not be achieved. These patients were returned to the ICU, where they were subjected to monitored skeletal traction using 10 kg weight to begin with and second hourly increase of weight by 2 kg up to a maximum of 24 kg. Skeletal traction was attempted for a period of 48 h during which time serial check x-rays were taken at four hourly intervals [Figure 5].

**Figure 6:**
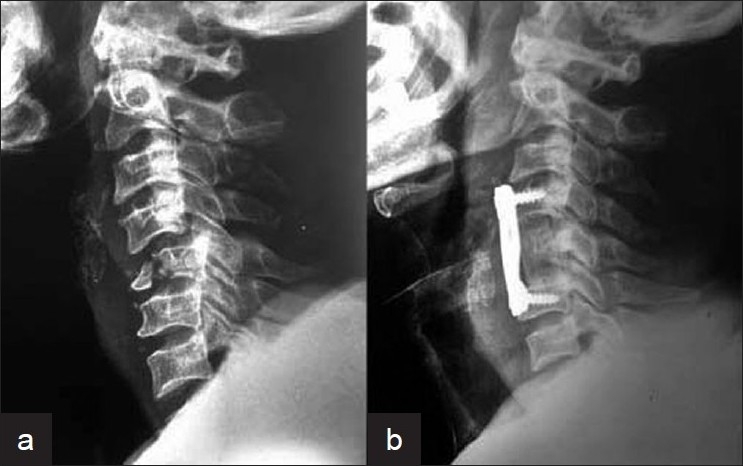
X-ray cervical spine (lateral view) showing (a) C5 fracture with subluxation. (b) C5 fracture with subluxation treated with anterior corpectomy, fusion with fixation (21 months post-operative)

In 25 patients reduction of subluxation was achieved with skeletal traction. These patients were then subjected anterior iliac graft fusion and fixation with cervical plate and screws [Figure 6].

**Figure 7:**
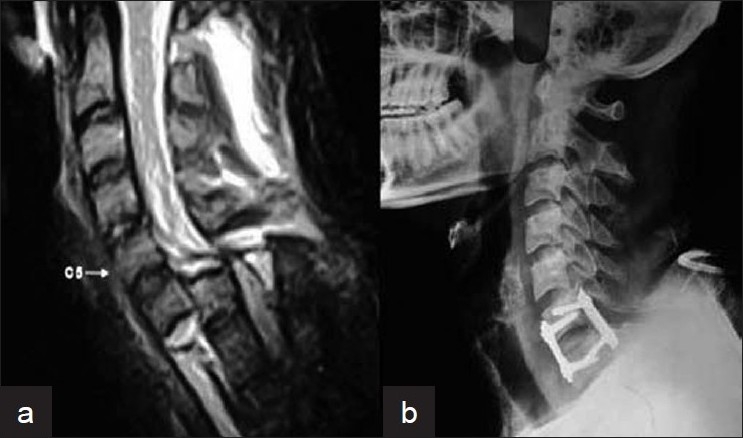
(a) Pre-op sagittal MRI T2W image of C6-C7 dislocation in whom reduction could not be achieved despite on-table and post-operative traction. (b) Post-operative X-ray of cervical spine (lateral view) showing posterior facetectomy and global fixation

Eleven patients in whom reduction could not be achieved despite 48 h of skeletal traction were subjected to posterior facetectomy and reduction following which posterior fixation was done (sublaminar wires in three patients, lateral mass screws and plate in four patients, and transpedicular screws with plate fixation in four patients) [Figure 7]. This was followed by the patient being turned supine and anterior iliac graft and fixation with cervical plate and screws were done.

All cases requiring surgery were subjected to surgery directly after admission except in 12 patients in whom the general medical condition prevented them from undergoing an immediate surgery. These 12 patients were initially stabilized using skeletal tongs till they could be taken up for surgery.

Post-operative X-rays and detailed neurological examination was carried out in all patients. Check x-rays and neurological evaluation was documented at 6 weeks, 3 months, and 6 months following surgery. Post-operative neurological grading was also done as per the ASIA scale.

X-rays were used to assess instability (angular of >11° or >3.5 mm translation on flexion-extension films)^3^ and fusion as indicated by radiological evidence of crossed bony trabeculation, uniform radiodensity of the graft with the body, and lack of segmental mobility. Evidence of uniform radiodensity of the graft with adjacent bone at both the ends was taken to be complete fusion. Uniform density of the bone graft interface at one end with or without crossing trabeculations at the other end with no evidence of instability on flexion and extension films was taken as partial fusion. Graft height was assessed for evidence of collapse.

All patients were mobilized within a week except in cases where the patient-associated injuries or neurological status prevented mobilization. Philadelphia collar was used for 6 weeks in all cases.

## RESULTS

Postoperative neurological and radiological follow up for a minimum period of 6 months (mean=1 year 3 months; range: 6 months to 3 years 2 months) was possible in 143 operated cases. One hundred and twenty-six patients showed good fusion (88.1%), while the remaining 17 (11.8%) had partial fusion. None of the operated cases showed evidence of instability on flexion-extension films [Tables 1 and 2].

**Table 1:** Summary of surgically treated patients

Mechanism of injury in surgically treated cases	Involvement of facets		Mode of fixation and fusion		
	Bifacetal dislocation	Unifacetal dislocation	On table reduction achieved+AF	Reduction with post-decompression traction+AF	Facetectomy and global fixation
Sciwora (19)			19	NA	
Burst compression fracture (18)			18	NA	
Distraction hyperextension (57)	41	16	50	5	2
Distraction hyperflexion (27)	17	10	24	2	1
Translation rotation alone (27)	19	8	17	10	0
Translation rotation with end plate/tear drop fracture (43)	29	14	27	8	8
Total=191			155	25	11

AF - Anterior fusion, NA - Not applicable

**Table 2:** Fusion rated of surgically treated patient with a minimal follow up of 6 months

Fusion in surgically treated cases	Complete	Partial
Sciwora (13)	12	1
Burst compression fractures (12)	11	1
Distraction hyperextension (39)	36	3
Distraction hyperflexion (16)	14	2
Rotation/translation (20)	18	2
Rotation/translation with end plate/tear drop fractures (43)	35	8
Total=143	126 (88.1%)	17 (11.8%)	

**Figure 8:**
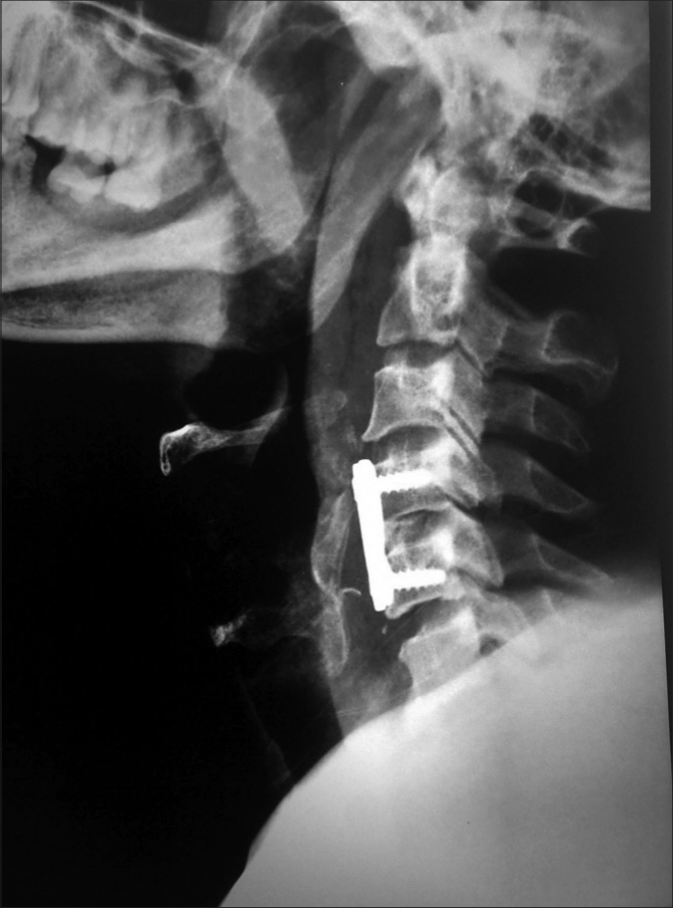
Follow up radiograph of cervical spine (lateral view) of a case of C4-C5 retrolisthesis showing good fusion and alignment obtained by anterior decompression, fixation, and fusion

Of the 70 patients with translation/rotation injuries, associated end-plate fracture and/or tear drop/quadrangular fractures were noted in 43 cases. In eight of these cases reduction could not be achieved and global fusion was done, while in the remaining cases anterior fixation was sufficient. Six out of eight treated with global fusion showed complete fusion while two patients had partial fusion. Of those treated with anterior fusion alone, 54 patients had complete fusion [Figure 8] while 8 patients had partial fusion [Table 2].

**Figure 9:**
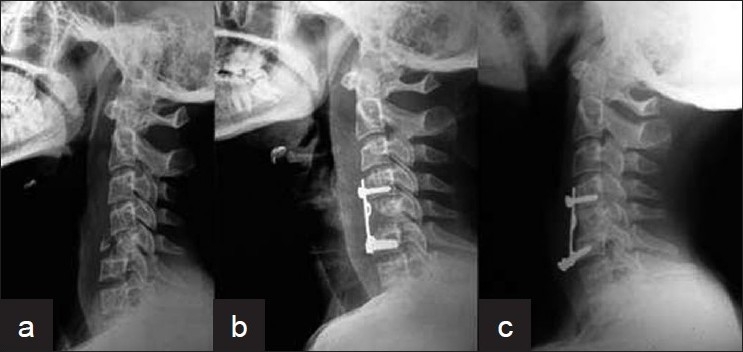
X-ray cervical spine lateral view showing (a) Patient with fracture C5 with retrolisthesis. (b) Post-operative X-ray of the patient treated with anterior corpectomy, fusion, and fixation. (c) Follow up film shows good fusion, inferior screws have backed out, and graft height is reduced

Reduction of graft height was noted in 28 patients. Two of these had multiple level corpectomy and four cases had undergone multiple level discectomy. There was no instance of breakage of the plates; however eight patients had loosening and backout of screws (12 screws) [Figure 9]. Importantly seven of these patients were noted to have reduction of graft height. Four patients underwent re-exploration to tighten the screws, in two patients’ fusion had already being achieved, and hence the implants were removed, while two patients were unwilling for re-exploration.

Four patients developed superficial wound infection at the cervical site including one patient who underwent global fusion. Graft site infection was noted in three patients. All seven patients responded to antimicrobial therapy. Two patients developed transient recurrent laryngeal nerve palsy and one patient developed a trachea-esophageal fistula which healed with conservative management. Fifteen patients needed a tracheostomy for their management.

On post-operative neurological examination, 23 patients showed improvement in neurological function following surgery, importantly four patients who had been quadriplegic (ASIA grade A) for more than 48 h showed significant neurological recovery post-operatively–ASIA grade E in one case and ASIA grade D in three cases. Four patients developed worsening of neurological deficits after surgery of these two patients with C_4_-C_5_ dislocation and quadriplegia developed loss of pre-existing elbow flexion; in the other two cases including one patient who required posterior facetectomy and global fusion there was worsening of motor power by two grades in the immediate post-operative period; both patients showed improvement to pre-operative status at 3 months.

Eight patients expired in the immediate post-operative period (1 week) of these patients two patients associated with polytrauma-developed pulmonary embolism and two other patients developed pulmonary consolidation with pneumonitis the remaining patients developed respiratory difficulty probably secondary to ascending cord edema. Two other patients developed delayed respiratory infection and septicemia and expired on the 24^th^ and 47^th^ post-operative day, respectively [Table 3].

**Table 3:** Mortality chart

Age	Type of injury	Level of injury	Pre-op status	Associated injuries/disease	Cause of death	Time of death	Surgery performed
31 yr	Bi-facetal dislocation	C_5_-C_6_ dislocation	Quadriplegia	Multiple long bone fractures	Pulmonary embolism	3^rd^ post-op day	Anterior discectomy, reduction, fusion+fixation
18 yr	Bi-facetal dislocation with tear drop fracture	C_5_ #, C_5_-C_6_ dislocation	Quadriplegia	Multiple long bones + pelvis fractures	Pulmonary embolism	5^th^ post-op day	C_5_ corpectomy, fusion+fixation
36 yr	Bi-facetal dislocation	C_5_-C_6_ dislocation	Quadriplegia	(L) hemothorax + lung contusion	Respiratory failure 2° to lung trauma	4^th^ post-op day	Discectomy, reduction fusion+fixation
19 yr	C_4_	Burst fracture	Quadriplegia	B/L hemothorax	Respiratory failure 2° to lung trauma	3^rd^ post-op day	C_4_ corpectomy fusion+fixation
38 yr	Bi-facetal dislocation	C_4_-C_5_	Quadriplegia	DM+HT	? Ascending cord edema	2^nd^ post-op day	Anterior discectomy, reduction, fusion+fixation
59 yr	Bi-facetal dislocation	C_4_-C_5_	Quadriplegia	Nil	? Ascending cord edema	1^st^ post-op day	Anterior discectomy, reduction, fusion+fixation
31 yr	Bi-facetal dislocation	C_3_-C_4_	Quadriplegia	Nil	? Ascending cord edema	3^rd^ post-op day	Anterior discectomy, reduction, fusion+fixation
29 yr	Burst fracture with dislocation	C_5_ #, C_4_-C_5_	Quadriplegia	Nil	? Ascending cord edema	6^th^ post-op day	C_5_ corpectomy, fusion+fixation
22 yr	Bi-facetal dislocation	C_4_-C_5_	Quadriplegia	Nil	Respiratory infection + septicemia	47^th^ post-op day	Anterior discectomy, reduction, fusion+fixation
37 yr	Burst fracture	C_5_	Quadriplegia	DM+HT	Respiratory infection + septicemia	24^th^ post-op day	C_5_ corpectomy, fusion+fixation

#: Fracture, DM: Diabetes mellitus, HT: Hypertension

## DISCUSSION

Some studies have reported no permanent worsening after close reduction of an undecompressed spine in a neurologically intact alert patient. While others have reported neurological deterioration after primary closed reduction.^4^–^8^ These studies also show the incidence of traumatic disc prolapsed to be 18% before and 56% after closed reduction. The application of skeletal traction after decompression avoids the risk of herniation of a disc or bony fragment into the spinal canal and in addition the disc excision facilitates body separation which helps achieve reduction.

Manual traction used to achieve reduction can generate forces equivalent to 30-40 kg, presenting a high risk of stretch and deformity of the cord leading to worsening of neurological deficits;^9^,^10^ hence in our opinion excessive or prolonged manipulation in cases where on table reduction is not achieved is best avoided.

In 36 cases where after reasonably vigorous attempts with manual traction, reduction was not achieved, we reversed our patients and subjected the conscious patients to progressively increasing traction loads (up to 24 kg) under close monitoring for up to 48 h. Various studies have shown that in an alert patient fairly high traction loads could be applied safely.^2^,^10^,^11^ We too feel that subjecting the patient to fairly high traction loads under close monitoring is safe and could avoid the need for a posterior procedure in a significant number of cases where on table reduction was not possible (25/36=69.4%).

Posterior fixation provides a more rigid fixation compared to anterior stabilization; however, the rigidity provided by anterior fixation is significantly higher than in an intact motion segment.^12^ The anterior approach in addition provides direct access to the cord and the compressive elements and has a lower incidence of post-surgical kyphosis.^13^

Global stabilization was advocated in patients with retrolisthesis, facet dislocation, and shear injuries.^14^ More recently, global fusion has been recommended in translation rotational injuries associated with end-plate fractures and burst or tear drop fractures;^12^,^15^ however good fusion rates (85-90%) have been achieved following anterior fusion and fixation in flexion, distraction, and translation rotational injuries.^12^,^16^

We have hence preferred to use the anterior approach as our primary mode of access and proceeded to posterior facetectomy and global fusion only in cases where reduction cannot be achieved even with post-decompression traction. With this modified protocol posterior facetectomy and global fusion was needed only in 11/154 cases (7.1%) of distraction or rotational/translational injuries.

Fusion rates of 90-100% in a single-level corpectomy fusions and 70-100% in two-level corpectomy fusions have been reported by Cheng *et al*.^17^ Johanson *et al*.^15^ reported mechanical failure in two thirds of cases with end-plate fracture associated with facet fracture, subluxations, or dislocations. We had a complete fusion rate of 88.1% (126/143) and a partial fusion of 11.8% (17/143) which is similar to the previous reported literature. However, eight patients had backing out of screws and notably seven of these had reduction of graft height. We feel that reduction of graft height post-operatively may contribute significantly to failure of stabilization; hence we use a slightly oversized graft (about 10% higher following a corpectomy and 20% following a discectomy) to compensate for the same. The slight distraction achieved by an oversized graft also helps to maintain alignment in patients who have retrolisthesis. As cages have not been used for the patients in the study, we cannot comment on the advantages of using them. We have not used compression plates in our study; however, Epstein *et al*. have reported 97% fusion rates with compression plates which minimizes graft shielding and facilitates graft compression.^18^

As in most studies, the neurological outcome^5^,^19^ was largely determined by the neurological grade in which the patient presented. However, four patients who presented with complete quadriplegia (ASIA grade A) for more than 48 h showed significant improvement after surgery. On discharge one patient was ASIA grade E, while three were ASIA grade D; hence we feel that the advantages of decompression prior to reduction and stabilization should be offered to all patients regardless of their neurological status. This delayed recovery differs from the findings of Manson *et al*. where no neurological improvement was noted in patients who were completely paraplegic for more than 48 h after injury.^20^

One of the shortcomings of our study is the lack of comparison with other techniques. We have tried to bring out an unbiased evaluation of one method of fixation in this study and not used it to compare it with other techniques. The neurological and radiological evaluation at regular post-operative intervals was as per standardized set protocols in order to minimize the bias.

## CONCLUSION

Anterior decompression and stabilization in our opinion could be used in most cases of unstable cervical injury regardless of the mechanism. The need for posterior or global stabilization is not mandatory in cases with translation rotation injuries or retrolisthesis as advocated by other studies.

In a significant number of patients in whom on-table reduction is not achieved, post-decompression satisfactory alignment can be achieved with monitored traction post-operatively. This obviates the risk of subjecting the patient with a decompressed unstable spine to a prone position under anesthesia and the need for a posterior procedure.

The proposed algorithm provides an easily replicable, saf, e and cost-effective method of managing subaxial cervical injuries.
